# Performance feedback enhances test-potentiated encoding

**DOI:** 10.3389/fnbeh.2023.1100497

**Published:** 2023-04-20

**Authors:** Petra Ludowicy, Pedro M. Paz-Alonso, Thomas Lachmann, Daniela Czernochowski

**Affiliations:** ^1^Cognitive and Developmental Psychology Unit, Center for Cognitive Science, University of Kaiserslautern-Landau (RPTU), Kaiserslautern, Germany; ^2^Cognitive Neuroscience Unit, Center for Cognitive Science, University of Kaiserslautern-Landau (RPTU), Kaiserslautern, Germany; ^3^BCBL, Basque Center on Cognition, Brain and Language, San Sebastian, Spain; ^4^IKERBASQUE, Basque Foundation for Science, Bilbao, Spain; ^5^Centro de Investigación Nebrija en Cognición (CINC), Universidad Nebrija, Madrid, Spain; ^6^University of Leuven, Leuven, Belgium

**Keywords:** reinforcement learning, episodic memory, testing effect, test-potentiated learning, feedback, retrieval

## Abstract

**Introduction:**

Long-term memory retention is enhanced after testing compared to restudying (testing effect). Notably, memory retrieval further improves when correct-answer feedback is provided after the retrieval attempt (test-potentiated encoding–TPE).

**Methods:**

To evaluate whether explicit positive or negative feedback further enhances memory performance beyond the effect of TPE, in two experiments additional explicit positive or negative performance-contingent feedback was presented before providing correct-answer feedback. After an initial exposure to the full material, 40 participants learned 210 weakly associated cue-target word pairs by either restudying or testing (Experiment 1). Depending on the accuracy of the retrieval attempt, the tested word pairs were followed by positive or negative performance feedback (50%) or no feedback (50%). Irrespective of the type of repetition, trials were followed by a restudy opportunity. Participants returned to perform a final cued-recall test (Day 2).

**Results:**

Final test results replicated the testing effect (better memory performance for tested compared to restudied items). Explicit performance feedback in addition to correct-answer feedback increased retrieval performance, but only on Day 2. This pattern of results was replicated in Experiment 2 in an independent sample of 25 participants. To assess the specific effects of learning history, we also examined retrieval accuracy and reaction times during repetition cycles: Explicit feedback improved retrieval for material successfully encoded in the initial study phase (consistent positive feedback) as well as for material learned during the repetition phase (mixed positive and negative feedback).

**Discussion:**

Performance feedback improves learning beyond the effects of retrieval practice and correct-answer feedback, suggesting that it strengthens memory representations and promotes re-encoding of the material.

## Introduction

Learning and memory are typically investigated in two separate research fields even though they can be conceived as two sides of the same coin. Research investigating learning historically focuses on the (co-)occurrence and timing of several stimuli (classical conditioning) and their influence on later behavior based on reinforcement or punishment (operant conditioning), thus providing insights into the biological mechanisms. However, more complex types of learning tied to higher cognitive functioning, such as episodic long-term memory, can be more susceptible to the influence of other internal and external cognitive factors (e.g., attention, monitoring, and motivation). To date, only a few investigations integrated both topics, for instance by implementing conditioning into memory tasks (for review see Miendlarzewska et al., 2016) and thus, have emphasized their relation and mutual influence. In line with these studies, the present study investigated the role of positive and negative performance-contingent feedback for episodic learning by implementing principles of reinforcement into a cued recall paradigm.

Episodic memory paradigms investigating the *testing effect* provide a good basis to implement feedback. More precisely, memory tests are a powerful, although not widely used, technique to improve later memory retrieval (see Roediger and Butler, 2011). Generally, in testing effect paradigms an (1) initial study phase is followed by a (2) repetition phase, in which participants practice the material by either testing or restudying it, followed by a (3) final memory test. Increased memory performance is observed after testing compared to restudying (i.e., testing effect, see Roediger and Butler, 2011; Rowland, 2014). This effect was replicated many times with different material (e.g., Karpicke and Roediger, 2008; Weinstein et al., 2011; Butler et al., 2013; Pastötter and Bäuml, 2016) and in different settings (e.g., Rohrer et al., 2010; Vojdanoska et al., 2010). While testing enhances memory performance, receiving correct answer feedback after a test further boosts the mnemonic advantage of the testing effect (e.g., Carrier and Pashler, 1992 or Cull, 2000). Another presentation of the full material (i.e., restudy opportunity) serves as correct answer feedback, i.e., *test-potentiated encoding* (TPE; Arnold and McDermott, 2013; Van den Broek et al., 2016; Rickard and Pan, 2018). For instance, Butler et al. (2007, 2008) reported improved memory recall when correct answer feedback was provided after a test, compared to no restudy opportunity. Thus, a retrieval attempt maximizes the benefit from a subsequent presentation of the full material (Arnold and McDermott, 2013), potentially because the learner can correct errors and validate correct responses given with low confidence (Butler and Roediger, 2007, 2008).

Prior research primarily suggests two possible mechanisms as the source of the TPE. First, as originally suggested by Roper (1977), the modulation of attentional resources might be a key factor for TPE. When provided with correct answer feedback, participants can shift their attention to selectively re-encode relevant material (Roper, 1977; Van den Broek et al., 2016), specifically for instances in which the initial retrieval was incorrect. This process can be enhanced by providing external feedback (Ludowicy et al., 2019). On the other hand, the second idea proposes that memory representations are extended and enriched during the reactivation of the encoding and retrieval context (e.g., Vestergren and Nyberg, 2014). It is still an open question whether error-detection processes and/or the re-encoding process underlie the effect of TPE.

In order to evaluate the underlying mechanisms, we propose to dissociate error detection and re-encoding of the material, occurring at the same time during correct answer feedback. A temporal separation was implemented by providing explicit performance-contingent feedback before presenting correct answer feedback. More precisely, some responses in the practice phase were immediately followed by positive or negative feedback depending on prior recall success. Hence, participants would not need to verify their behavior themselves based on the correct answer feedback and could focus on re-encoding the material during the subsequent presentation of correct answer feedback instead. Both mechanisms previously discussed as the origin of TPE might benefit from performance feedback. On the one hand, previous studies suggest that rewards can modulate attentional orientation (see e.g., Miendlarzewska et al., 2016). Note that recent literature on performance feedback revealed its rewarding effects even without monetary gains (e.g., Daniel and Pollmann, 2010; see also review by Ferdinand and Czernochowski, 2018). On the other hand, performance feedback might enrich the memory representation since this additional mnemonic information can be integrated into the already existing memory structure and thus strengthen the memory trace. As suggested by Van den Broek et al. (2014), strong memory traces are thought to be retrieved more rapidly and with more confidence.

Some prior studies combined performance feedback and correct answer feedback, although indirectly, for instance by presenting the correct answer in green or red font (Butler et al., 2007; Ernst and Steinhauser, 2012). In contrast, only few studies aimed to disentangle the differences between these types of feedback (Roper, 1977; Pashler et al., 2005). For instance, Roper (1977) and Pashler et al. (2005) compared memory recall boosted by either performance feedback, correct answer feedback or no feedback. While Roper (1977) found a beneficial effect of performance feedback, Pashler et al. (2005) did not. These mixed findings suggest that performance feedback might enhance episodic memory under certain conditions only: Both studies did not combine performance feedback with a restudy opportunity, preventing re-encoding of the material, and hence, one of the potential mechanisms possibly underlying TPE. To the best of our knowledge, only one study (Fazio et al., 2010) combined performance feedback and correct answer feedback by replicating the previously mentioned experiments (Roper, 1977; Pashler et al., 2005) while adding another review cycle. This additional review cycle might serve as a block-wise and delayed correct answer feedback (Rowland, 2014). Fazio et al. (2010) reported a selective performance feedback benefit for low-confidence correct responses. In line with Pashler et al. (2005), they did not find a general memory enhancement due to performance feedback, although the reinforcing quality of performance feedback has been previously suggested to influence learning (Jou and Foreman, 2007). However, firm conclusions on potential effects of performance feedback seem premature at this point and may for instance depend on the number of repetition cycles and delay to final test.

One possible reason for the small magnitude of performance feedback effects in the study by Fazio et al. (2010) is that initial testing with performance feedback and repeated presentation of the full material was performed block-wise. This delay between performance feedback and correct answer feedback might prevent effective attention shifting and focusing. Hence, the present study aimed to disentangle the effects of performance feedback and correct answer feedback, using only a small temporal delay. While performance feedback provides explicit information for error-detection, correct answer feedback offers an opportunity for implicit error-detection as well as reencoding. The presentation of performance feedback immediately before the correct answer feedback temporally separates these two processes that occur at the same time if only correct answer feedback is presented. In line with this idea, we propose that separating the processes of error-detection and re-encoding the material might help to dissociate the mechanisms of the test potentiated encoding. Furthermore, these mechanisms might provide insights into the relation between learning by reinforcement on more complex types of learning. We expected to (1) replicate the standard testing-effect with our paradigm and stimuli. In addition, we predicted that (2) memory performance in the final test increases when additional performance feedback is provided. Performance feedback might help to shift attention and enrich the memory representation. Enriched memory representations may be particularly relevant for trials in which correct, but weak memory traces have been formed during initial encoding, whereas shifts of attention are specifically useful if no retrievable memory traces have been formed initially. Hence, we evaluated whether the history of retrieval success on Day 1 influences memory performance on Day 2. More specifically, we assessed if (3) the beneficial effect of performance feedback is larger for items with mixed retrieval history compared to items consistently retrieved correctly. Finally, we explored the temporal course of memory performance by assessing (4) if the feedback effect increased over time.

## Experiment 1

### Materials and methods

#### Participants

Forty-four healthy German native speakers volunteered in Experiment 1. All participants were right-handed, reported to have normal or corrected-to-normal vision and had no red-green deficiency (i.e., they were able to perceive the colored feedback symbols). The sample size was determined based on previous studies investigating the testing effect (e.g., Butler et al., 2007 or Fazio et al., 2010). None of them reported any history of psychological or neurological diseases, or acute use of psychoactive substances. In addition, participants reported normal sleep patterns, including the night before final testing. Following information about the procedure, all participants gave written informed consent and received payment or course credit for their participation. Three participants had to be excluded from data analyses due to low memory performance (2 SDs lower than the mean) and one participant due to technical problems during data acquisition. Data from a final sample of 40 participants (25 females; mean age = 24.5 years; SD = 2.7 years) were analyzed.

#### Material

A total of 210 German translations of weakly associated cue-target word pairs (e.g., *feather-duck*; *towel-soap*) were selected from Nelson et al. (2004) database. All word pairs were controlled for forward strength (FSG; FSG > 0.04), backward strength (BSG; BSG > 0.04), mediated strength (MSG; MSG > 0.04), and overlapping strength (OSG; OSG > 0.05). The assignment of word pairs to conditions was randomized across participants.

#### Procedure

**FIGURE 1:**
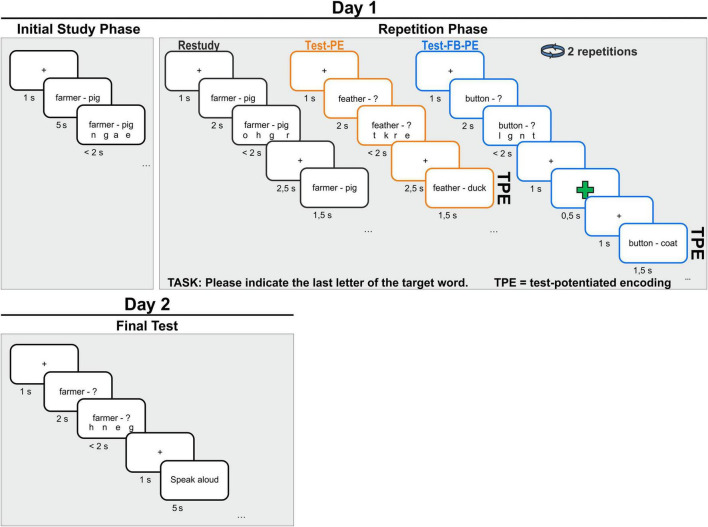
Main schematic procedure of Experiment 1.

Experiment 1 was divided into two sessions spaced about 24 h apart (see Figure 1). In the first session, participants were exposed to 210 word pairs in an initial study phase. Afterward, participants were asked to retrieve 140 word pairs, followed by another exposure to the full word pair (TPE). Half of these retrieval practice items were additionally followed by explicit binary feedback (i.e., green plus or red minus sign) contingent upon prior retrieval performance. A green plus sign was presented to indicate a correct performance and a red minus sign for incorrect performance. In other words, participants were asked to retrieve the target word for each of the 70 word pairs in the Test-FB-PE condition, and received explicit performance feedback before another exposure to the full word pair. For the remaining 70 word pairs, participants were asked to retrieve the target word, but received no performance feedback before the second exposure to the full word pair (Test-PE condition). Note that in this condition, the performance during the retrieval attempt can be inferred indirectly from the correct answer provided as a restudy opportunity. Finally, participants were asked to merely restudy 35 word pairs (Restudy condition) without a prior retrieval attempt. These 175 word pairs were repeated during two repetition cycles, whereas the remaining 35 word pairs were only presented once in the initial study phase (Control condition).

One day later, participants returned to the lab for a final test. The experiment was conducted on a computer using Presentation (Neurobehavioral Systems, Berkeley, CA, USA) to control stimulus presentation and timing.

#### Session 1

In the first session, participants were instructed to intentionally learn as many word pairs as possible during the initial study phase as well as afterward during the repetition phase, in which they restudied or retrieved the target words twice (repetition cycle 1 and 2). In the initial study phase, all word pairs (e.g., German: *Feder-Ente/*English: *feather-duck*) were presented individually for 7 s at the center of the screen. Participants were informed that they would later be tested on the target words (e.g., *duck*) upon the presentation of the cue words (e.g., *feather*). After 5 s of stimulus presentation, four response letter options (e.g., “l r s o”) appeared below the word pair. Participants were instructed to select the last letter of the target word from the four letters by pressing the corresponding key on a keypad with the left- or right-hand index or middle finger [e.g., “e” for *Ente* (*duck*), i.e., 4-alternative forced choice recognition]. We familiarized participants with this response procedure during the initial study phase. Each trial was preceded by a 1 s fixation cross. Short breaks of 60 s were carried out every 50 trials.

Following the initial study phase, in the repetition phase participants either retrieved or restudied 175 word pairs with the instruction to memorize them. Word pairs were randomly assigned to one of the following three conditions: (I) In the *Restudy* condition, participants were presented with 35 full word pairs (i.e., cue and target word together), one at a time at the center of the screen, for a maximum of 4 s. After 2 s, 4 letters appeared underneath the word pair. One of these letters represented the last letter of the target word. As in the initial learning phase, participants were asked to indicate this letter via button press. After the response, a fixation cross appeared for 2.5 s. (II) In the *Test-PE* condition, participants were only presented with the cue word of 70 word pairs and a question mark at the position of the target word, for a maximum of 4 s. Then, they were asked to retrieve the target word from memory. Next, the response options appeared after 2 s with the same response procedure. In this condition, a fixation cross appeared for 2.5 s after the participants gave a response. (III) The *Test-FB-PE* condition was the same as the Test-PE condition, with additional performance-contingent positive or negative feedback for 500 ms, followed by a fixation cross of 1 s. For all conditions, the full word pair (i.e., cue and target word) was presented for another 1.5 s after each practice trial, and participants were instructed to restudy the word pair. Thus, as illustrated in Figure 1, word pairs were either restudied, tested or tested with subsequent performance feedback. Critically, participants saw the correct answer for the same amount of time in the Test-PE and Test-FB-PE conditions to provide equal opportunity to re-encode the material.

After all word pairs were practiced in one of the 3 conditions (i.e., 1st repetition cycle), participants repeated these procedures in the 2nd repetition cycle to assess if the effect of feedback depends on a long consolidation phase or not. All words were assigned to the same conditions in both repetition cycles. Brief pauses of 60 s were carried out every 50 trials. Responses were scored correct only within 2 s after the response letters appeared. Before leaving the lab, participants were instructed not to rehearse the word pairs at home.

#### Session 2

After about 24 h (Experiment 1 *M* = 24 h; *SD* = 1.3 h), participants returned to the lab to perform the second session in which all 210 word pairs were tested by presenting the cue word with a question mark for 4 s. The order of cue words was randomized, and rests of 60 s were carried out every 50 trials. Similar to the Test-PE condition of the repetition phase, participants were asked to retrieve the target word from memory. The response procedure was the same as at the previous day (4-alternative forced choice of the last letter of the target word; response options appeared below the cue word after 2 s). Afterward, another fixation cross was presented for 1 s. Finally, participants were requested to speak aloud the full word pairs (i.e., cued recall, pronouncing the cue word presented on the screen for 5 s and the target word retrieved from memory).

#### Analysis

The paradigm was designed to investigate accuracy differences in the final test and repetition cycles. Recall accuracy in the repetition phase was rated based on the letter-indication task and at final test based on verbal responses. In addition, reaction times (RTs) were assessed by calculating the median RT for each participant from the onset of the presentation of the response options until the response was provided by button press in the letter-indication task as potential index for memory strength. RTs of the repetition phase and the final test were measured in the same way. RTs faster than 200 ms were excluded from the present analyses. The analyses of the repetition phase were restricted to correct responses since there were not enough incorrect trials for some conditions. In contrast to some previous studies on the testing effect, we used a within-participant manipulation in order to minimize variance caused by individual differences. Four questions were investigated: (1) Can the testing effect + TPE be replicated? (2) Does performance-contingent feedback further boost memory performance on Day 2? (3) Does positive and negative feedback during the repetition cycles differently influence memory performance in the final test? (4) Does feedback already influence memory performance on Day 1 (during the repetition cycles)? All four research questions were investigated by examining both recall accuracies and RTs.

First, in order to address research questions 1 and 2, recall accuracy and RT results from the final test (Day 2) were investigated as a function of the repetition *Conditions* (Control, Restudy, Test-PE, Test-FB-PE) by means of a repeated measures Analyses of Variance (ANOVA) (trial numbers: Control: *M* = 6.1, *SD* = 4.0; Restudy: *M* = 19.9, *SD* = 7.4; Test-PE: *M* = 54.6, *SD* = 10.5; Test-FB-PE: *M* = 56.1, *SD* = 9.1). Repeated within-subject contrasts were used to investigate different levels of main effects (Control vs. Restudy, Restudy vs. Test-PE, Test-PE vs. Test-FB-PE). These contrasts were planned beforehand, according to the predictions and based on previous evidence (Roper, 1977; Pashler et al., 2005; Fazio et al., 2010; Van den Broek et al., 2014; Racsmány et al., 2018).

Second, in order to directly assess the effects of retrieval history (Day 1) on final test performance (research question 3), results from the final test were conditionalized based on the retrieval success during the repetition cycles (only possible for the Test-PE and Test-FB-PE conditions) and analyzed with a 2 × 2 ANOVA with the factors *Condition* (Test-PE vs. Test-FB-PE) and *Retrieval History* (early success vs. late success; trial numbers: early Test-PE: *M* = 37.2, *SD* = 11.5; late Test-PE: *M* = 17.4, *SD* = 4.5; early Test-FB-PE: *M* = 36.5, *SD* = 11.0; late Test-FB-PE: *M* = 19.5, *SD* = 5.6). Word pairs that were correctly remembered in both repetition cycles were correctly encoded during the initial study phase and hence assigned to early retrieval success, whereas all others were assigned to late retrieval success. Note that feedback was always contingent on individual performance, hence trials receiving positive and negative feedback differ in both, original memory performance and type of feedback presented subsequently. Word pairs with fewer than two valid responses during the repetition phase could not be categorized and hence had to be excluded from this analysis (0,001%).

Finally, in order to evaluate the temporal course of TPE (research question 4), recall accuracies and RTs collected in the first and second repetition cycle (Day 1) were analyzed with a 2 × 2 ANOVA with the factors *Repetition cycle (cycle* 1 vs. cycle 2) and *Condition* (Test-PE vs. Test-FB-PE; trial numbers: cycle 1 Test-PE: *M* = 42.7, *SD* = 8.4; cycle 2 Test-PE: *M* = 57.4, *SD* = 7.9; cycle 1 Test-FB-PE: *M* = 42.5, *SD* = 8.7; cycle 2 Test-FB-PE: *M* = 57.8, *SD* = 7.7). The RT analysis was based on trials for which participants provided a correct response in the letter-indication task, since there were only few incorrect responses, especially in repetition cycle 2. For all analyses, Greenhouse–Geisser correction was employed where appropriate.

### Results

#### Testing effect and feedback–Recall accuracy and RT in the final test (research questions 1 and 2)

**FIGURE 2:**
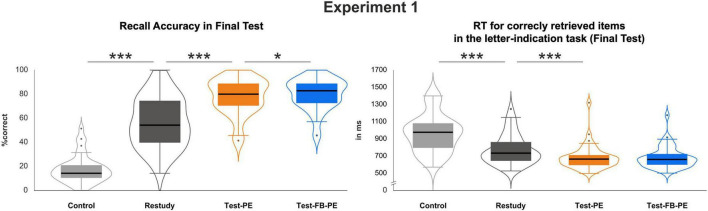
Recall accuracy and RT results at final test as a function of repetition condition for Experiment 1. Note that the control condition (i.e., items studied only once) in Experiment 1 is associated with both lowest recall accuracy and highest RTs. The intervention benefit is evident in terms of both increased accuracies and decreased RTs. *p < 0.05 and ***p < 0.001.

For Experiment 1, an ANOVA with the factor *Condition* (Control, Restudy, Test-PE, Test-FB-PE, see Figure 2; Table 1) with repeated contrasts revealed increased recall accuracy on Day 2 for Test-PE items compared to Restudy items, *F*(3, 117) = 513.10, *p* < *0.001*, η_*p*_^2^ = 0.93 (Test-PE vs. Restudy: *p* < 0.001, η_*p*_^2^ = 0.77; see Figure 2). Likewise, memory performance was lower for items in the Control condition compared to the Restudy condition items (Restudy vs. Control: *p* < 0.001, η_*p*_^2^ = 0.87). In addition to these typical testing effect results, word pairs tested with performance feedback on Day 1 (Test-FB-PE items) showed an increased memory performance on Day 2 compared to the Test-PE condition (Test-FB-PE vs. Test-PE: *p* = 0.024, η_*p*_^2^ = 0.12).

**TABLE 1:** Summary of results for research question 1–4 of experiment 1 (±SD).

	Recall success	RT
Control	18% (±11.5)	961 ms (±217)
Restudy	57% (±21.1)	779 ms (±172)
Test-PE	78% (±15.0)	687 ms (±143)
Test-FB-PE	80% (±12.9)	685 ms (±129)
Research question 1: control vs. restudy	*p* < 0.001; η_p_^2^ = 0.87	*p* < 0.001; η_p_^2^ = 0.44
Research question 1: restudy vs. test-PE	*p* < 0.001; η_p_^2^ = 0.77	*p* < 0.001; η_p_^2^ = 0.28
Research question 2: test-PE vs. test-FB-PE	*p* = 0.024; η_p_^2^ = 0.12	*p* = 0.805; η_p_^2^ = 0.002
Research question 3		
Early test-PE	89% (± 9.9)	669 ms (± 150)
Late test-PE	65% (± 18.8)	770 ms (± 151)
Early test-FB-PE	90% (± 8.2)	660 ms (± 115)
Late test-FB-PE	69% (± 16.7)	734 ms (± 183)
Research question 4		
Cycle 1 test-PE	63% (± 12.1)	798 ms (± 159)
Cycle 2 test-PE	84% (± 11.5)	668 ms (± 87)
Cycle 1 test-FB-PE	62% (± 12.6)	816 ms (± 170)
Cycle 1 test-FB-PE	84% (± 10.9)	662 ms (± 93)

A similar pattern was found for RTs, *F*(3, 114) = 42.50, *p* < 0.001, η_*p*_^2^ = 0.53 (see Figure 2; Table 1), with considerably faster responses for word pairs in the Test-PE condition compared to the Restudy or Control condition (Test-PE vs. Restudy: *p* < 0.001, η_*p*_^2^ = 0.28; Restudy vs. Control: *p* < 0.001, η_*p*_^2^ = 0.44). No RT difference was found for items that were tested with performance feedback compared to the ones tested without feedback (Test-FB-PE vs. Test-PE: *p* = 0.805, η_*p*_^2^ = 0.002).

#### Effects of early vs. late retrieval success on final test performance–Recall accuracy and RTs as a function of initial response accuracy (research question 3)

**FIGURE 3:**
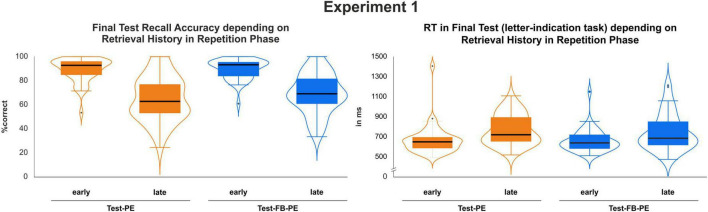
Accuracy and RT results at final test as a function of retrieval history (at Day 1) (early vs. late success) and repetition practice (Test-PE vs. Test-FB-PE).

A 2 × 2 ANOVA with the factors *Condition* (Test-PE vs. Test-FB-PE) and *Retrieval History* (early vs. late success) revealed a main effect of *Retrieval History*, *F*(1, 39) = 155.73, *p* < 0.001, η_*p*_^2^ = 0.80 (see Figure 3, left, and Table 1), reflecting enhanced memory performance in the final test following early retrieval success during the repetition phase. In addition, a main effect of *Condition* indicated an improvement in memory performance for Test-FB-PE items compared to Test-PE items, *F*(1,39) = 4.69, *p* = 0.037, η_*p*_^2^ = 0.11. The interaction did not reach statistical significance [*F*(1,39) = 2.02, *p* = 0.163, η_*p*_^2^ = 0.05].

The same pattern of results was found for RT: a main effect of *Retrieval History*, *F*(1,39) = 31.22, *p* < 0.001, η_*p*_^2^ = 0.45 (see Figure 3, right, and Table 1) revealed faster responses in the final test if word pairs were retrieved correctly in both retrieval attempts. Moreover, a main effect of *Condition* indicated faster responses for Test-FB-PE [*F*(1,39) = 4.25, *p* = 0.046, η_*p*_^2^ = 0.10]. The interaction did not reach statistical significance [*F*(1,39) = 1.15, *p* = 0.291, η_*p*_^2^ = 0.03].

#### Effects of performance feedback and repeated exposure during the repetition phase–Accuracy and RT (research question 4)

**FIGURE 4:**
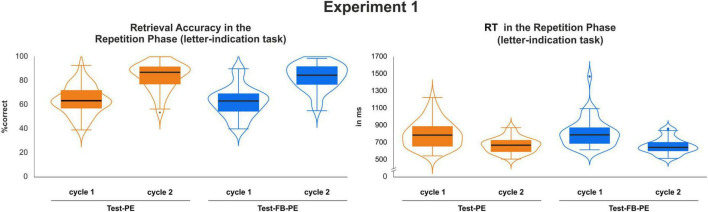
Retrieval accuracy and RT results during repetition cycle 1 and 2 with as a function of repetition practice (Test-PE vs. Test-FB-PE).

A 2 × 2 ANOVA investigating retrieval accuracy in the repetition phase (i.e., selecting from four response options) with the factors *Condition* (Test-PE vs. Test-FB-PE) and *Repetition Cycle* (1st cycle vs. 2nd cycle) revealed a main effect of *Repetition Cycle*, with higher memory performance after repeated exposure to the material, *F*(1,39) = 377.45, *p* < 0.001, η_*p*_^2^ = 0.91 (see Figure 4, left, and Table 1). Memory performance in the second repetition cycle was higher compared to the first test in the first cycle. In line with prior literature, no effect of performance feedback was found. Neither the main effect of *Condition* [*F*(1,39) < 1, *p* = 0.561, η_*p*_^2^ = 0.01], nor the interaction reached statistical significance [*F*(1,39) < 1, *p* = 0.547, η_*p*_^2^ = 0.01].

Analyzing RT of correctly retrieved items revealed a main effect of *Repetition Cycle, F*(1,39) = 59.53, *p* < 0.001, η_*p*_^2^ = 0.60, (see Figure 4, right, and Table 1) confirming faster responses in the second compared to the first repetition cycle. In sum, independent of the presence of performance feedback, memory performance increased with repetition while RTs decreased. Again, neither the main effect of *Condition* [*F*(1,39) < 1, *p* = 0.539, η_*p*_^2^ = 0.01], nor the interaction reached statistical significance [*F*(1,39) < 1, *p* = 0.205, η_*p*_^2^ = 0.04].

## Discussion experiment 1

Experiment 1 assessed whether performance-contingent feedback in combination with subsequent correct-answer feedback modulates the testing effect, by comparing participants’ speed and accuracy in the repetition phase (Day 1) and the final test (Day 2). Our results are in line with previous findings in terms of enhanced memory following retrieval practice (i.e., testing effect) and a benefit of correct-answer feedback (i.e., TPE). When opportunities to re-encode are kept constant (TPE, c.f. Rowland, 2014), retrieval practice enhances later memory performance (Butler et al., 2007). Specifically, compared to restudying, prior testing resulted in an increase in accuracy from 57 to 79%, as opposed to 18% accuracy after a single learning cycle (control condition). We extend previous findings by demonstrating that explicit performance feedback further enhances TPE (by 2%) after a delay of one day. In addition, analyzing final test recall and RT depending on Day 1 performance suggests a beneficial effect of performance feedback independent of early or late retrieval success on Day 1.

Most studies investigating the testing effect and TPE have examined overt retrieval accuracy (e.g., Roediger and Karpicke, 2006; Butler et al., 2007). Participants in our paradigm indicated their response by selecting one out of four alternatives (see also Wing et al., 2013; Van den Broek et al., 2014; Wirebring et al., 2015; Pastötter and Bäuml, 2016; Wiklund-Hörnqvist et al., 2017). This particular test format enabled us to also evaluate RTs in addition to memory performance, as lower memory strength may lead to slower response times since retrieval is more effortful (Wixted and Rohrer, 1993; Van den Broek et al., 2014; Racsmány et al., 2018). Critically, the requirement to select the last letter ensured that participants needed to recall the word, if only covertly. However, it is conceivable that the last letter may be relatively salient and potentially support cued recall for a subset of items. To exclude this possibility, in Experiment 2, participants were required to select the 3rd rather than the last letter of the target word.

In line with the results reported by Van den Broek et al. (2014), we found smaller RTs for previously tested compared to restudied items and in addition, restudying improved retrieval speed compared to a single learning opportunity. Performance feedback modulated RT when retrieval success on Day 1 was included in the analysis. Two underlying mechanisms could explain this effect. On the one hand, retrieval mechanisms may become increasingly efficient with retrieval practice. In line with Racsmány et al. (2018), faster responses in the second compared to the first repetition cycle for correctly remembered words support this idea. On the other hand, an improvement in monitoring processes may lead to enhanced re-encoding during TPE as participants already received information about the correctness of their response. In line with this thought, we found faster responses for both, material learned in the initial learning phase as well as material learned in the repetition cycles receiving additional support through performance feedback.

Taken together, Experiment 1 provides evidence that principles of reinforcement learning can modulate episodic memory performance. Since the performance benefit of explicit performance feedback in the Test-FB-PE condition was relatively modest compared to correct answer feedback only (Test-PE condition), we examined whether this performance difference would replicate in an independent sample.

## Experiment 2

Experiment 2 included a total of 26 German native speakers (14 females; mean age = 23.5 years; SD = 3.0 years) who volunteered to participate in this experiment. Data from one participant was excluded from these study analyses due to low memory performance (i.e., 2 SDs lower than the mean). As this study aimed at replicating the results of Experiment 1, the sample size was determined based on previous studies investigating the testing effect with comparable trial numbers per condition (e.g., Pastötter and Bäuml, 2016; Wiklund-Hörnqvist et al., 2017; Ludowicy et al., 2022).

### Material

In Experiment 2, a total of 180 weakly associated German cue-target word pairs were presented. Stimuli were controlled in a similar way to the ones used in Experiment 1, and the assignment of the word pairs to conditions in these experiments was also randomized across participants.

### Procedure

Experiment 2 closely matched the procedure of Experiment 1. However, no control condition was included to increase the amount of trials per condition (i.e., 60 word pairs were used for Restudy, Test-PE and Test-FB-PE conditions, respectively). Hence, Research Questions 1 and 2 were addressed by investigating recall accuracy and RT results from the final test (Day 2) as a function of the repetition *Conditions* (Restudy, Test-PE, Test-FB-PE) by means of a repeated measures ANOVA (trial numbers: Restudy: *M* = 29.9, *SD* = 13.7; Test-PE: *M* = 42.8, *SD* = 10.3; Test-FB-PE: *M* = 44.9, *SD* = 10.1).

Moreover, there were slight modifications in the timing of each trial: in the repetition phase of Experiment 2, performance feedback was presented for 1 s in the Test-FB-PE condition and the fixation cross was presented for 3 s in the Restudy or Test-PE conditions. Correct answer feedback was presented for 2 s in Experiment 2 as compared to 1.5 s in Experiment 1. In Experiment 2, participants responded with the fingers of their right hand and participants were instructed to report the third letter of the target word by pressing the corresponding key as compared to the last letter in Experiment 1. All other aspects of the experimental procedure, including the timing of stimulus presentation stayed the same. Similar to Experiment 1, the delay between Session 1 and 2 was also 24 h (*SD* = 1.3 h) in Experiment 2.

## Results

**FIGURE 5:**
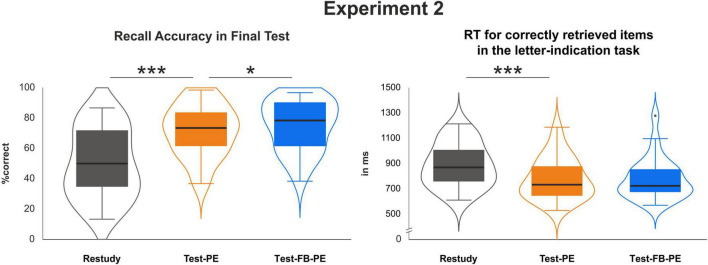
Recall accuracy and RT results at final test as a function of repetition condition for Experiment 2. The intervention benefit is evident in terms of both increased accuracies and decreased RTs. *p < 0.05 and ***p < 0.001.

Similar to Experiment 1, an ANOVA with the factor *Condition* (Restudy, Test-PE, Test-FB-PE) revealed improved memory performance for Test-PE compared to restudied items [*F*(2, 48) = 54.66, *p* < 0.001, η_*p*_^2^ = 0.70]: In line with the typical testing effect, memory for previously tested items was considerably higher compared to restudied (Test-PE vs. Restudy: *p* < 0.001, η_*p*_^2^ = 0.70; see Figure 5). Critically, additional performance feedback increased memory even further (Test-PE-FB vs. Test-PE: *p* = 0.046, η_*p*_^2^ = 0.16). Furthermore, RT results revealed a similar pattern [*F*(2,48) = 13.18, *p* < 0.001, η_*p*_^2^ = 0.35] with faster responses for Test-PE compared to restudied items (Test-PE vs. Restudy: *p* < 0.001, η_*p*_^2^ = 0.39). However, across both experiments, no reliable RT difference between Test-PE and Test-FB-PE items was observed (Test-PE-FB vs. Test-PE: *p* = 0.837, η_*p*_^2^ = 0.002; see Figure 5; Table 2).

**TABLE 2:** Summary of results for research question 1 and 2 of experiment 2 (±SD).

	Recall success	RT
Restudy	50% (±22.9)	888 ms (±173)
Test-PE	71% (±17.1)	783 ms (±166)
Test-FB-PE	75% (±16.9)	779 ms (±166)
Research question 1: restudy vs. test-PE	*p* < 0.001; η_p_^2^ = 0.70	*p* < 0.001; η_p_^2^ = 0.39
Research question 2: test-PE vs. test-FB-PE	*p* = 0.046; η_p_^2^ = 0.16	*p* = 0.837; η_p_^2^ = 0.002

## General discussion

By providing explicit performance feedback immediately prior to a restudy opportunity, we assessed the role of performance feedback for enhancing the testing effect. We thus extend previous findings by demonstrating that explicit performance feedback further enhances TPE after a delay of 1 day. Although the benefit of providing explicit performance feedback in addition to correct answer feedback was modest, it was still reliably observed across two experiments. In the following, we will discuss (I) how our findings clarify previously inconsistent results regarding the role of performance feedback (II) under which conditions we expect a larger impact on memory performance and (III) how RT data largely neglected in previous studies can provide additional insight into the underlying mechanisms of feedback enhanced learning.

While our findings are in line with the literature in terms of TPE, the potential role of performance feedback may have been underestimated in prior studies. One factor concerns the timing of the experimental paradigm. The cognitive processes associated with performance feedback and a restudy opportunity (correct answer feedback) may depend on temporal contiguity of both factors, which is lost when a delay between performance feedback and correct answer feedback is introduced. A block-wise retrieval practice followed by a block-wise restudy opportunity presumably decreases the influence of performance feedback on TPE. In contrast to the study by Fazio et al. (2010), we addressed this point by presenting the correct answer feedback immediately after the performance feedback. A second important factor addresses the delay between initial learning and final test. A larger testing effect is reliably observed when a delay of at least 24 h is introduced between the repetition phase and the final test (Roediger and Karpicke, 2006; Rowland, 2014). Consequently, shorter delays might underestimate the testing effect along with the factors that can modulate it. In our data, we can compare the beneficial effect of performance feedback on TPE at the end of Day 1 with the results at the final test on Day 2. In line with prior results (Fazio et al., 2010), we did not observe any differences between items tested with or without feedback after the second practice cycle on Day 1. Hence, benefits in memory accuracy due to the combination of performance feedback and correct answer feedback appeared only after a delay of 24 h.

The main difference between the two TPE conditions in the present study is that performance feedback can be inferred indirectly at the time of the restudy opportunity in the Test-PE condition, whereas it is explicitly provided in the Test-FB-PE condition. Using cognitive resources to infer prior performance indirectly may not be very effective, as it would distract participants from using the restudy opportunity. By contrast, providing explicit feedback immediately before the restudy opportunity enables participants to selectively direct attention to *relevant* information as suggested by Roper (1977) and facilitate error-detection processes. Additionally, positive and negative feedback might enrich the memory representations (e.g., Vestergren and Nyberg, 2014). In the present paradigm, providing explicit feedback may be particularly helpful for items that received negative feedback (i.e., the ones that have not been sufficiently encoded, in some instances also raising the need to override a highly confident but incorrect response). Also items retrieved correctly, but given with low confidence may benefit from the restudy opportunity (i.e., items that require a strengthening of an existing memory trace). Future studies may include confidence ratings or subjective judgments of learning to disentangle both instances more explicitly since greater accuracy at monitoring one’s learning during study was associated with higher levels of retention (Agarwal et al., 2008; Dunlosky and Rawson, 2012).

While the beneficial effects of providing explicit performance feedback in addition to correct-answer feedback (i.e., Test-FB-PE vs. Test-PE) appear to be modest, the present work highlights that these effects are reliable. We expect to see an even larger impact of explicit feedback when retrieval accuracy is lower, for instance for material which is more difficult to retrieve or by increasing the delay to the final test on Session 2. Regardless of the numerical magnitude, the underlying mechanisms supporting these beneficial effects of adding explicit performance feedback are still under debate. We predicted that performance feedback could either promote attention shifts toward the most relevant–previously incorrect–information (Ludowicy et al., 2019) or reinforce specific correct behavior and thus further enhance memory representations for initially correctly memorized items. In line with this idea, we analyzed final test results as a function of retrieval success during the repetition phase (Day 1), assuming that enhanced memory retrieval in the final test could vary depending on the timing of successful encoding. Material learned during the initial study phase benefits from consistent positive feedback during the repetition phase, whereas material learned during the repetition phase might take advantage of a combination of both, negative and positive feedback. Hence, learning should improve more when the mechanisms of positive and negative feedback are combined. In favor of this assumption, later memory retrieval and RT results of the present study implicate a beneficial effect of performance feedback for both, material learned at an early stage as well as material learned during the repetition cycles. Item difficulty was not affecting this result since items were randomly assigned to one of the conditions. Consequently, the present results imply that both mechanisms may be involved in TPE.

The present study revealed that performance feedback can facilitate learning from TPE. Nonetheless, the present findings need to be replicated with different learning materials or modulated by the delay between the two feedback types. Providing performance feedback based on recall success instead of the results during the letter-indication task might strengthen the performance feedback effect as well, which might provide the possibility to investigate individual differences in learning supported by feedback. Furthermore, behavioral measures only provide limited knowledge about specific mechanisms and the results highly depend on the design of the experiment, and other interpretations are conceivable. Neuroimaging techniques such as electroencephalography (EEG) or functional magnetic resonance imaging (fMRI) offer additional insight into the temporal and structural neuronal processes underlying the testing effect (e.g., Ernst and Steinhauser, 2012; Pastötter and Bäuml, 2016; Van den Broek et al., 2016; Wiklund-Hörnqvist et al., 2021). In detail, different types of feedback may modulate neural activity of brain areas involved in memory and feedback processing and consequently affect long-term learning (Van den Broek et al., 2016). In our own work using a closely related paradigm (Ludowicy et al., 2022), we examined the neural processes during successful memory retrieval with fMRI revealing an increase in functional coupling of memory- and feedback-related brain areas due to additional performance feedback which suggests that error-detection as well as re-encoding process are modulated.

## Conclusion

In order to uncover the mechanisms underlying TPE, we assessed whether combining performance-contingent feedback with correct answer feedback further improves final memory retrieval. Our results support this claim across two independent samples of participants, providing compelling evidence that principles of reinforcement learning can modulate episodic memory performance. In addition, the combination of retrieval accuracy and RT results demonstrates that both negative and positive feedback contribute to the beneficial feedback effect. Hence, performance-contingent feedback may be a helpful tool to promote episodic learning, as long as it is provided in close temporal proximity to the initial retrieval attempt and a subsequent restudy opportunity.

## Data availability statement

The raw data supporting the conclusions of this article will be made available by the authors, without undue reservation.

## Ethics statement

The studies involving human participants were reviewed and approved by the Ethics Committee of the University of Kaiserslautern. The patients/participants provided their written informed consent to participate in this study.

## Author contributions

PL, PP-A, and DC designed the research. PL collected and analyzed the data. PL and DC wrote the manuscript. PP-A and TL provided substantial additions or revisions of the manuscript. All authors contributed to the interpretation of the analysis and approved the submitted version of the manuscript.
